# Plenary Symposium

**DOI:** 10.1111/ene.70673

**Published:** 2026-06-26

## Sunday, June 28 2026

## Presidential Symposium (Named Lectures)

## PLEN02_3

### Navigating the translational arc for childhood movement disorders

#### M. Kurian

##### University College London, United Kingdom

## PLEN02_5

### What research and patients may teach us: Parkinson's disease—A history and perspective of learning

#### D. Berg

##### Department of Neurology, Universitätsklinikum Schleswig Holstein, Campus Kiel, Kiel, Germany

## PLEN02_7

### Non‐invasive neuromodulation in neurology

#### J. Rothwell

##### Department of Motor Neuroscience and Movement Disorders, University College London, London, United Kingdom

## PLEN02_9

### Progress in gliomas: From histology to molecular biology and from surgery to precision therapies

#### R. Soffietti

##### Department of Neuro‐Oncology, University and City of Health and Science Hospital (Torino), Torino, Italy

## Tuesday, June 30 2026

## Highlights and Breaking News

## PLEN03_92

### Jes Olesen Award Lecture: Peripherin: A novel early diagnostic and prognostic plasmatic biomarker in Amyotrophic Lateral Sclerosis

#### A. Bombaci

##### Vita‐Salute San Raffaele University, Milan, Italy

**Background:** Motor neuron diseases (MND) are heterogeneous and complex neurodegenerative disorders. Biomarkers could facilitate early diagnosis, prognosis determination, and patient stratification. Among the most studied biomarkers are neurofilaments, with peripherin (PRPH), a specific type predominantly expressed in peripheral nervous system, gaining attention. To date, no studies have evaluated PRPH in human plasma.

**Methods:** sandwich‐ELISA was used to quantify plasma peripherin from 120 MND (100 ALS, 4 PMA, 15 PLS), 73 MND‐mimics and 38 healthy‐controls (HCs). Plasma was collected at diagnosis or some months earlier. 41 ALS were evaluated longitudinally. ALSFRSr, MRC, spirometry, genetic tests, disease progression rate (PR), blood examinations, and neuropsychological tests were performed. Statistical analyses included Kruskal‐Wallis, Mann‐Whitney, Cox‐regression, and Kaplan‐Meier curves.

**Results:** Plasma PRPH levels differed significantly among groups (*p* < 0.0001), showing higher values in MND participants than MND mimics and HCs. Moreover, PRPH levels were elevated in PLS compared with HSP patients (*p* = 0.0001). Differences persisted after adjusting for age and sex. ROC curve demonstrated that PRPH discriminated MND from MND mimics (AUC = 0.85). Elevated PRPH correlated positively with ALSFRSr and lower motor neuron index, while inversely with disease progression rate. Higher PRPH levels at the beginning of the disease were associated with longer survival.

**Discussion:** Plasma PRPH is raised in MND, particularly ALS, from the earliest stages, distinguishing MND from mimics and correlating with clinical parameters and survival. This suggests PRPH may reflect an endogenous response of lower motor neuron to injury. Further multicentre studies are required to refine the diagnostic and prognostic utility of PRPH in MND.

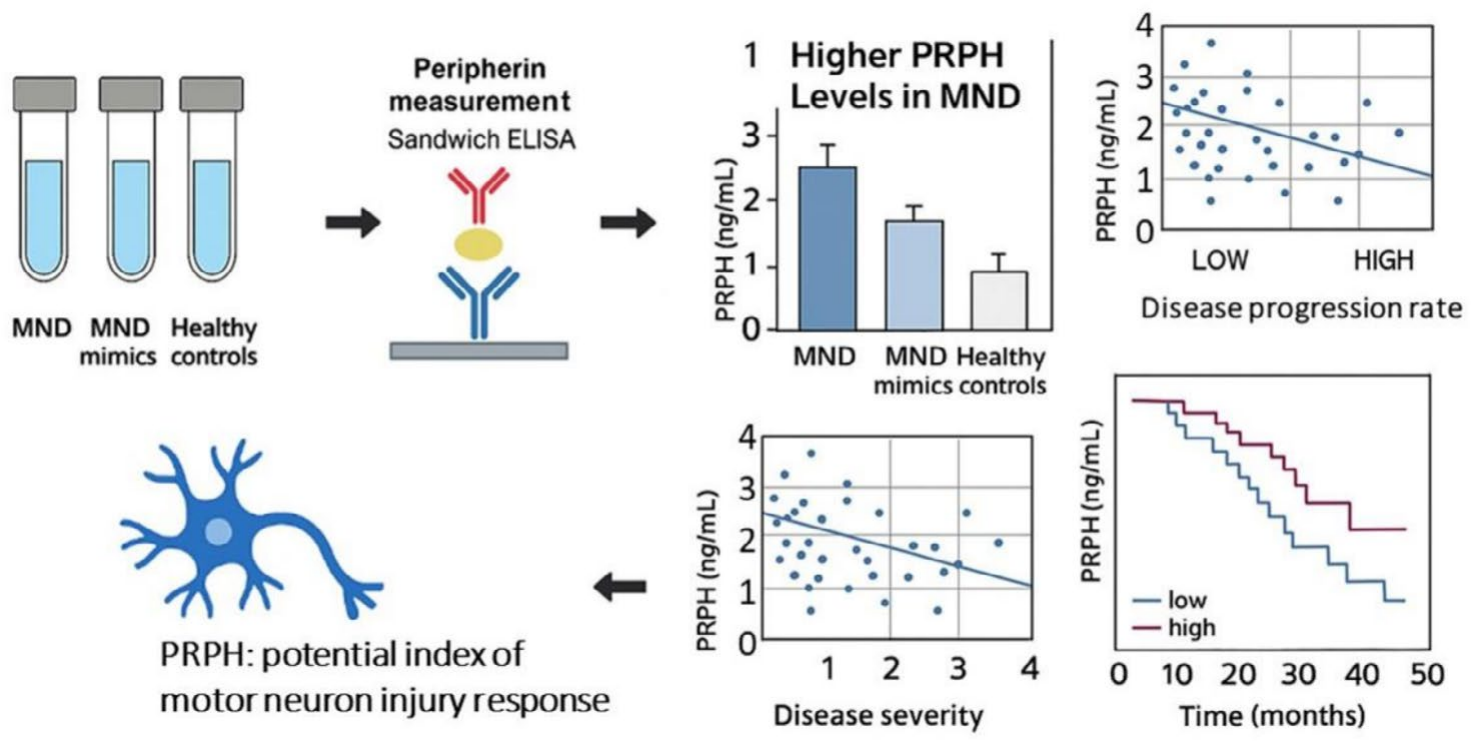

**Disclosure:** Nothing to disclose.

